# An Unusual Cause of Knee Mass: Osseous Hydatidosis

**DOI:** 10.7759/cureus.18556

**Published:** 2021-10-06

**Authors:** Cristina Carvalho Gouveia, Margarida Morais, Maria João Correia, Tiago Marques, Álvaro Pereira

**Affiliations:** 1 Internal Medicine Department, Hospital São Francisco Xavier, Lisbon, PRT; 2 Infectious Diseases Department, Hospital Santa Maria, Lisbon, PRT

**Keywords:** knee, bone, zoonotic infection, echinococcus granulosus, hydatidosis

## Abstract

Hydatid disease (hydatidosis) is a zoonotic infection caused by the larval stage of the parasitic tapeworm *Echinococcus granulosus *endemic in some sheep-raising areas. The liver and lungs are most commonly affected. Bone involvement (osseous hydatidosis) is distinctly uncommon, and its diagnosis and treatment can be challenging. We report a case of a 54-year-old male with right knee pain and edema and an extensive lesion on the femur; he was diagnosed with knee hydatidosis and was successfully treated with surgery and albendazole. This case reinforces the importance of the rare osseous hydatidosis as part of the differential diagnosis of bone lesions.

## Introduction

Hydatidosis is a parasitic infection caused by larval forms of cestode* Echinococcus* that is endemic in some sheep-raising areas of the Middle East, Central Asia, South America, and Mediterranean region [1-3]. Dogs and other carnivores, such as foxes, are the definitive hosts, while sheep, goats, and cattle are intermediate ones [4]. Humans get infected by contact with an infected animal or contaminated food [5]. After the parasite eggs are ingested, they reach the small intestine, and the majority of them (65%-75%) are carried to the liver [2-4]. Less frequently, larvae spread, across microcirculation, to other parts of the body, such as the bone, which occurs in 0.5%-2.5% of cases [3,4]. Because symptoms of osseous hydatidosis are nonspecific, its diagnosis is often made at late stages of the disease, being a high level of suspicion needed to attain it [6].

## Case presentation

**Figure 1:**
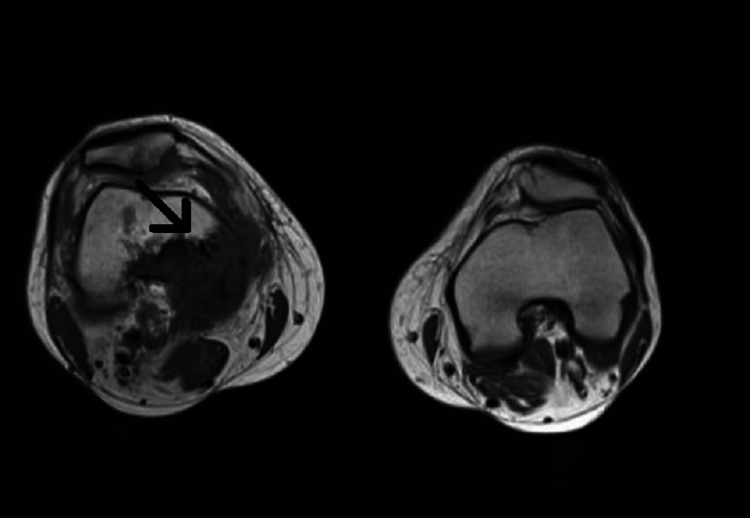
MRI of the knees Mass with cortical disruption, cystic areas, sclerosis, and adjacent soft tissue involvement

A healthy 54-year-old male, a resident in Portugal, presented to the outpatient Infectious Diseases Department with symptoms of right knee pain and edema for the past nine months. He reported contact with stray dogs weeks before the disease onset. There was no history of fever, constitutional symptoms, or trauma. The patient reported no genitourinary or gastrointestinal symptoms or gout. On physical examination, the knee was swollen and tender to palpation. Regarding laboratory tests, complete blood count, erythrocyte sedimentation rate, and routine biochemical tests, including C-reactive protein and lactate dehydrogenase, were in normal ranges. Computed tomography (CT) and magnetic resonance imaging (MRI) showed a 13-cm mass with cortical disruption affecting the lower portion of the diaphysis and metaphysis of the femur, with cystic areas, sclerosis, and adjacent soft tissue involvement (Figure 1).

**Figure 2:**
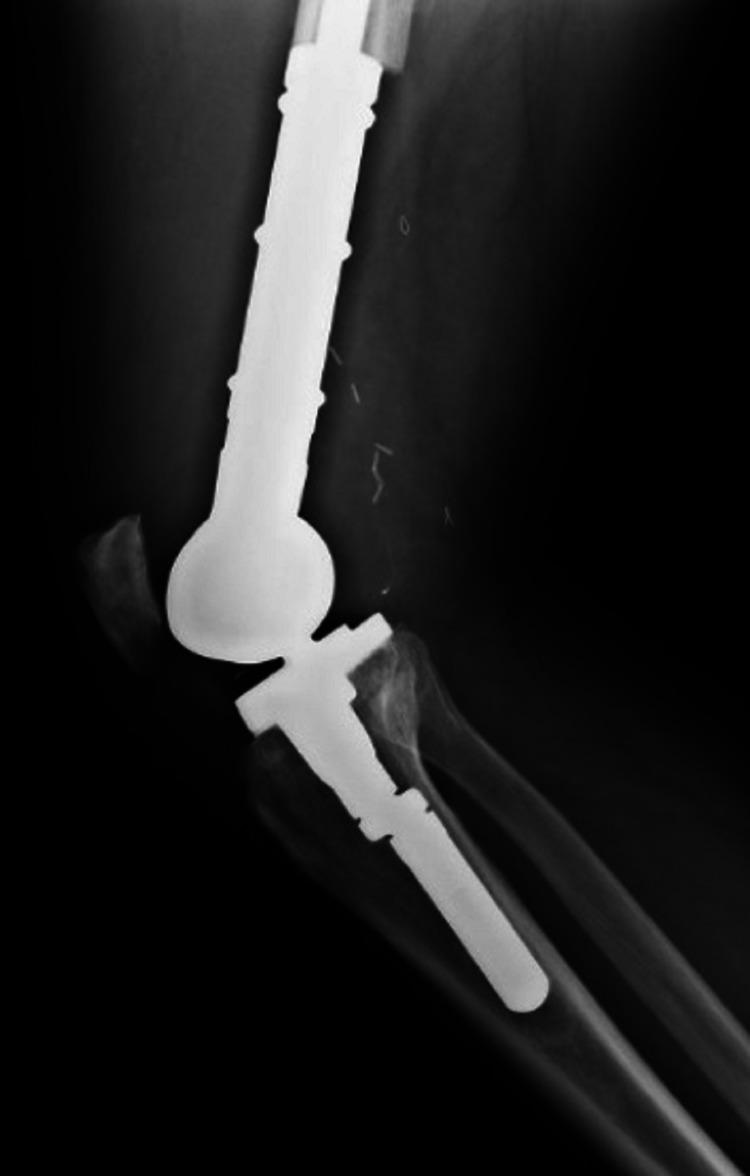
Lateral right knee radiography Knee replacement with prosthetic material

**Figure 3:**
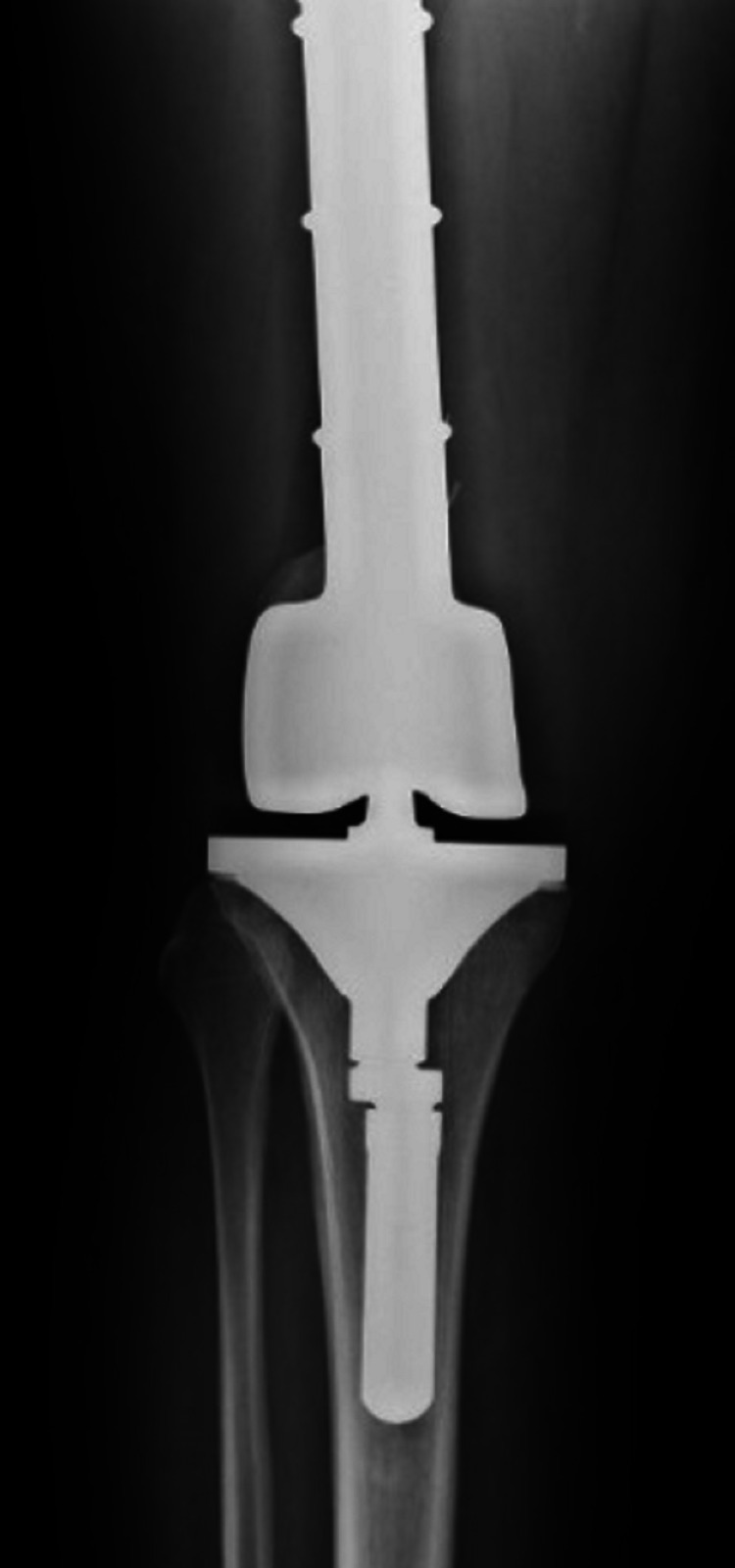
Anteroposterior right knee radiography Knee replacement with prosthetic material

At this point, the patient was referred to the Oncology Department, because osteosarcoma was considered the most probable diagnosis. The first biopsy showed no neoplastic cells and was otherwise normal. The second biopsy revealed an acellular and hyaline laminated membrane lined with small eosinophilic cells, suggestive of a hydatid cyst membrane. The serology of hydatid disease was positive, with a titer of 1/1280. A diagnosis of right knee hydatidosis, caused by *Echinococcus granulosus*, was made, and the patient was submitted to surgical resection and total knee replacement with insertion of prosthetic material (Figures 2 and 3).

Femur biopsy showed an extensive granulomatous reaction of the cortical and medullary bone that extended to the adjacent adipose and muscle tissues, composed of several acellular and hyaline laminated membranes (ectocysts) and rare scolices. The patient was started on albendazole 400 mg twice daily for two cycles of 28 days. No recurrence was determined clinically or radiologically in the three-month follow-up period, and there was also serological response, determined by the reduction of serological titers to 1/160.

## Discussion

In hydatidosis, humans become infected by consuming water or food contaminated with dog feces containing the parasite’s eggs [7]. After penetrating the small intestine mucosa, the embryos are carried to the liver by the bloodstream and, less frequently, to the lungs, where they form hydatid cysts [7]. Infrequently, larvae form cysts in other locations, such as the bone, where they cause a polycystic lesion that can be misdiagnosed as a bone tumor [7]. Osseous hydatidosis is a rare disease, accounting for 0.5%-2.5% of hydatidosis cases, and the spine is most commonly involved (35%-50%), followed by the pelvis (21%), femur (16%), and tibia (10%) [7]. Patients with osseous hydatidosis can refer pain and swelling or present with complications such as a fracture or cutaneous fistulization [8]. However, because symptoms are nonspecific, diagnosis is primarily based on roentgenographic findings [9]. CT and MRI can show cortical thinning, bone erosion and destruction, osteolytic lesions, osteosclerosis, pathological bone fractures, and soft tissue extension [3]. It is important to outline that bone destruction may cause significant osteolysis and can resemble a neoplastic lesion [3]. Serologic tests are also useful in the diagnosis and follow-up of patients [3]. A bone biopsy can assist in the diagnosis by showing the three layers of the cyst, namely, the outer pericyst, the middle acellular laminated membrane (ectocyst), and the inner germinal layer (endocyst), which produces the scolices that represent the larval stage [3]. The most appropriate treatment for osseous hydatidosis of long bones is surgical resection [10]. Adjuvant medical treatment with benzimidazole derivatives (mebendazole or albendazole) may be given before and after the surgery to control the disease, avoid systemic spreading, and prevent recurrences [10]. In Portugal, an endemic country, 13 people were diagnosed with hydatidosis in a three-year period, from 2013 to 2016 [11]. Our patient lived in this Mediterranean country; reported previous contact with stray dogs, which was consistent with fecal-oral transmission; and referred knee pain and swelling. However, the knee mass with cortical disruption observed in the MRI resembled osteosarcoma, and this disease was initially considered the most probable diagnosis. The anatomopathological examination showing an acellular laminated membrane and the positive serological test, in conjunction with the presence of an epidemiological context and observation of cystic areas and soft tissue involvement in the MRI, supported the diagnosis of osseous hydatidosis. The patient showed clinical and radiological improvement and reduction of serological titers after bone resection and two cycles of albendazole. A decision was made to interrupt this benzimidazole derivative and maintain patient follow-up for at least five years.

## Conclusions

In our case, although osseous hydatidosis was initially suspected because of the epidemiological context of the patient, the radiological findings were suggestive of a neoplastic etiology. Osseous hydatidosis is a rare disease that may be asymptomatic until a complication occurs. Additionally, imaging studies can resemble other entities, such as neoplasms and other inflammatory conditions. Because of the rarity of this disease and its lack of specific symptoms and radiological findings, a high index of suspicion is essential for its timely diagnosis and treatment. Therefore, osseous hydatidosis should be suspected in patients who present bone-related symptoms and show compatible radiological findings (namely, bone erosion and destruction, osteosclerosis, bone fractures, or soft tissue extension), particularly if they live in or travel to endemic areas.
